# Transcription quality control at the promoter-proximal checkpoint

**DOI:** 10.1101/gad.352973.125

**Published:** 2025-12-01

**Authors:** Daniel Blears, Jesper Q. Svejstrup

**Affiliations:** Center for Gene Expression, Department of Cellular and Molecular Medicine, University of Copenhagen, Copenhagen N 2200, Denmark

**Keywords:** DSIF, integrator, negative elongation factor 1, PAF1 complex, promoter-proximal pausing, RNA polymerase II, SPT5

## Abstract

Transcription of protein-coding genes by RNA polymerase II involves a characteristic pausing event downstream from transcription start sites. Such promoter-proximal pausing likely represents a transcription checkpoint, which ensures proper assembly of a fully functional RNAPII elongation complex that is capable of transcribing through the chromatin environment, with correctly modified nascent pre-mRNA. Failure to negotiate this promoter-proximal checkpoint results in termination of transcription and removal of RNAPII from the DNA. This review summarizes current knowledge on the mechanisms that regulate promoter-proximal pausing, maturation of the elongation complex, and the decision of whether to license RNAPII for elongation or prematurely terminate transcription.

## Abstract (precis)

In this review, Blears and Svejstrup elucidate the mechanisms of promoter-proximal pausing during eukaryotic transcription. They posit that promoter-proximal pausing serves as a critical checkpoint during transcription, allowing for the proper assembly and function of the RNA polymerase II elongation complex and the production of functional mRNA.

## Historical context

The RNA polymerase II (RNAPII) transcription cycle has typically for simplicity been broken down into the distinct stages of initiation, elongation, and termination. Briefly, initiation describes the recruitment of RNAPII to a gene promoter, melting of the DNA double helix, and generation of the first phosphodiester bond of the RNA, whereas elongation describes the productive synthesis of the mRNA transcript, until RNAPII reaches the end of the gene and transcription is terminated. However, already in the late 1970s, there were suggestions of important postinitiation events prior to the entry into productive transcript elongation. For example, it was discovered that 5,6-dichloro-1-β-D-ribofuranosylbenzimidazole (DRB) inhibits transcription at a step after initiation in both viral and cellular genes (Fraser et al. 1978; Sehgal et al. 1979; Tamm and Kikuchi 1979; Tweeten and Molloy 1981). Shortly thereafter, using in vitro labeling of transcripts in isolated nuclei, Chambon and colleagues (Gariglio et al. 1981) reported that RNAPII was only transcribing the 5′ end of the (inactive) β-globin gene in mature hen erythrocytes, whereas it transcribed the whole gene in immature erythrocytes where β-globin is expressed. It was later found that RNAPII also only transcribes the 5′ regions of, for example, c-myc, c-myb, and c-fos in mammalian cells in which these genes are largely inactive (Bentley and Groudine 1986; Eick and Bornkamm 1986; Nepveu and Marcu 1986; Bender et al. 1987; Fort et al. 1987) and the equivalent region of the uninduced *hsp70* heat-shock gene in the fruit fly (Gilmour and Lis 1986; Rougvie and Lis 1988, 1990). Further experimental analysis of this intriguing phenomenon by Lis, Groudine, Eick, and many others over the following years showed that RNAPII had indeed initiated transcription but appeared to pause or arrest after synthesis of only a relatively small number of nucleotides in the studied genes.

Independent of this progress, studies by Peterlin and colleagues (Kao et al. 1987) showed that the HIV-1 Tat protein appeared to exert its effect on HIV-1 gene expression at the level of transcript elongation. Subsequent biochemical analysis of the reaction indicated that the majority of elongation complexes generated by the HIV-1 promoter are not very processive and terminate transcription within the first several hundred nucleotides. However, upon addition of Tat, transcript elongation becomes much more processive. In a striking connection to previous work, transcript elongation in the presence of Tat was preferentially inhibited by DRB (Marciniak and Sharp 1991), indicating that this drug inhibits the generation of processive RNAPII elongation complexes. DRB had already several years previously been shown to inhibit a protein kinase in HeLa cell extract (Zandomeni and Weinmann 1984), and Tat protein was indeed found to associate with a protein kinase named Tat-associated kinase (TAK), which could phosphorylate the C-terminal repeat domain (CTD) of the largest RNAPII subunit (Herrmann and Rice 1995). Price and colleagues (Marshall et al. 1996) had independently isolated “positive transcription elongation factor b” (P-TEFb), which was also a CTD kinase, inhibited by DRB. Shortly thereafter, it was found that P-TEFb is identical to TAK and that P-TEFb/TAK is required for HIV-1 Tat transactivation (Yang et al. 1997; Zhu et al. 1997). Independent of this, DRB was also used by Handa and colleagues (Wada et al. 1998a; Yamaguchi et al. 1999) to isolate two protein complexes that play an important role in postinitiation regulation of mammalian transcription; namely, DRB sensitivity-inducing factor (DSIF) and negative elongation factor (NELF).

**Figure 1.**
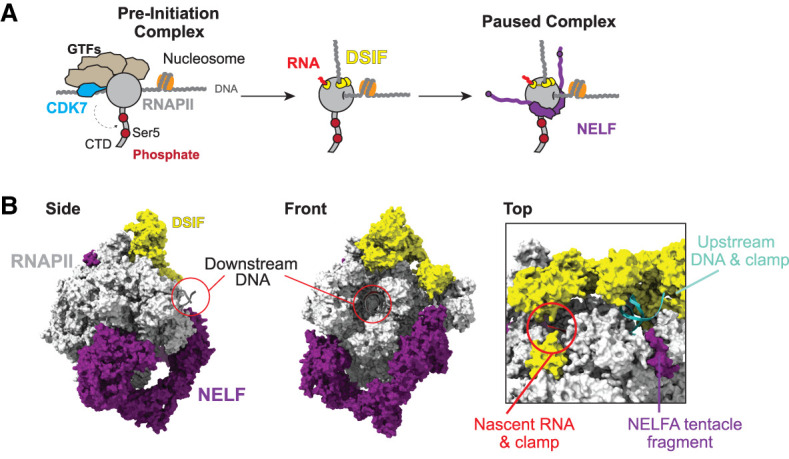
A paused elongation complex consisting of RNAPII, DSIF, and NELF. (*A*) Protein-coding gene transcription with recruitment of RNAPII to a gene promoter by general transcription factors and mediator, which form the preinitiation complex (PIC) and enable DNA melting and transcription initiation. The CDK7 kinase activity of TFIIH phosphorylates the RNA polymerase II CTD at serine 5 residues. DSIF binds to RNAPII, followed by NELF, resulting in a complex that is paused upstream of the first nucleosome. (*B*) Cryo-EM structure of the paused elongation complex (PDB: 8UHD). DSIF binds to the RNAPII surface and also to the upstream DNA duplex and the nascent RNA. DSIF forms DNA and RNA “clamps” and is an integral component of the paused and elongating transcription complexes.

With the advent of the genomics era, chromatin immunoprecipitation combined with microarrays or advanced sequencing (ChIP–chip and ChIP-seq, respectively) began to show that postinitiation regulation occurs at a sizeable proportion of genes in metazoan cells (see, e.g., Kim et al. 2005; Guenther et al. 2007; Muse et al. 2007; Zeitlinger et al. 2007), suggesting that such regulation might be more widespread than previously presumed. Over the last decade or more, a large variety of approaches has made it increasingly clear that so-called promoter-proximal pausing is indeed a key stage in the RNA polymerase II (RNAPII) transcription cycle on practically all metazoan protein-coding genes (Adelman and Lis 2012; Chen et al. 2018; Noe Gonzalez et al. 2021). Through the development of next-generation techniques such as GRO-seq and PRO-seq, we now know that upon initiation and transcription of 20–60 nt, RNAPII undergoes pausing, which depends on its association with DSIF and NELF in vitro (Fig. 1A; Wada et al. 1998b; Yamaguchi et al. 1999; Wu et al. 2003; Kwak et al. 2013; Vos et al. 2018b).

However, in spite of its frequent and seemingly fundamental nature, the biological role and purpose of promoter-proximal pausing has remained enigmatic. In some cases, as described above, pause escape into productive elongation appears to represent a rate-limiting step for transcription, suggesting that pausing can be a mechanism of transcription output regulation. Indeed, pausing at highly regulated, signal-responsive genes formed the basis for the hypothesis that the promoter-proximal pause functions as a major regulatory step, determining transcription levels (Adelman and Lis 2012; Core and Adelman 2019). Importantly, however, promoter-proximal pausing is detected at most genes in metazoans, including at constitutive “housekeeping” genes (Kwak et al. 2013; Fong et al. 2022). Furthermore, even in genes such as the heat-shock genes, which are switched on and off by control of promoter-proximal pausing and release, pausing occurs regardless of whether the gene is active or not (Tang et al. 2000). Such observations seem better in tune with a more general function for pausing in the RNAPII transcription cycle.

In this review, we focus on promoter-proximal pausing as an integral step of the eukaryotic RNAPII transcription cycle, which is required for the assembly of productive elongation complexes that are capable of traveling across the long genes of metazoans and ensure the production of functional mRNA.

## Promoter-proximal pausing as a checkpoint

The transition from transcription initiation to productive elongation appears to represent a distinct phase of the transcription cycle in which paused RNAPII recruits additional protein factors and matures into an “elongation complex.” Such an elongation complex must be equipped for, for example, passage through nucleosomes (Kujirai and Kurumizaka 2020), cotranscriptional processing of the nascent RNA (Shine et al. 2024), and dynamic post-translational modifications of histones and other chromatin components (Millán-Zambrano et al. 2022). Indeed, in order to successfully transcribe mammalian protein-coding genes, which are often hundreds of kilobases long, the elongation complex must be exceptionally stable and operate with high processivity and at high specificity; that is, without errors of nucleotide misincorporation. The fact that key proteins associated with paused transcription complexes such as NELF and Integrator are specific to metazoans and not found in yeast appears in tune with the idea that promoter-proximal pausing took on an increasingly important role with the evolution of longer genes.

Given that escape from this maturation phase and commitment to productive elongation can be interrupted through inhibition of certain kinases and phosphatases (more details are described below), these activities may be thought of as “licensing” RNAPII for productive elongation. The overall process of pausing and pause release may therefore in turn be thought of as a checkpoint, which ensures correct assembly of the elongation complex before RNAPII is committed to the process of transcribing the full length of the protein-coding gene. It seems reasonable to consider that this maturation phase, the “promoter-proximal checkpoint” (PPC), carefully evaluates the correct assembly before allowing mature RNAPII to progress. As with all checkpoints, a failure to pass it has consequences—in this case, premature termination of transcription. We discuss checkpoint execution and the possible reasons for its evolution below.

## Mechanism of promoter-proximal pausing

As mentioned above, the kinase inhibitor DRB was used to uncover or characterize several key players in the process of promoter-proximal pausing, including the cyclin-dependent kinase (CDK) complex P-TEFb (also known as CDK9/cyclin T), DSIF (also known as the Spt4/5 complex in yeast) (Decker 2021), and NELF. Together, DSIF and NELF inhibit transcription in vitro, but this effect is alleviated by P-TEFb, whose kinase activity induces commitment to the production of full-length transcripts (Wada et al. 1998b; Yamaguchi et al. 1999; Narita et al. 2003). Subsequent genome-wide studies have confirmed the role of P-TEFb, DSIF, and NELF as general regulators of transcription at the promoter-proximal pause, with perturbation of these factors resulting in different degrees of genome-wide alterations in RNAPII pausing and transcription dynamics (Aoi et al. 2020; Hu et al. 2021; Fong et al. 2022).

Purification of NELF and DSIF later revealed an association with Integrator (Stadelmayer et al. 2014; Yamamoto et al. 2014), a large, multisubunit complex containing various modules with RNA cleavage, protein phosphatase, and nucleic acid binding capabilities. Although originally isolated through its role in the processing (cleavage) of small nuclear RNA transcripts (Baillat et al. 2005), Integrator is now arguably better known for its involvement in the termination of RNAPII transcription at promoter-proximal pause sites. However, it is broadly involved in the termination of transcription that is unproductive; that is, not leading to the production of stable, polyadenylated RNAPII transcripts but instead generating unstable transcripts degraded by the nuclear exosome (Stadelmayer et al. 2014; Yamamoto et al. 2014; Lai et al. 2015; Skaar et al. 2015; Elrod et al. 2019; Tatomer et al. 2019; Huang et al. 2020; Zheng et al. 2020; Lykke-Andersen et al. 2021; Vervoort et al. 2021; Stein et al. 2022; Hu et al. 2023). Integrator appears to terminate transcription surprisingly frequently at the promoter-proximal pause and is a key player in the execution of the transcription checkpoint at the beginning of genes.

## The structural basis of pausing

Over the last several years, DSIF, NELF, and Integrator, typically in complex with RNAPII, have been visualized by cryo-electron microscopy (cryo-EM), which greatly informs our understanding of the structural basis of promoter-proximal pausing and release (Vos et al. 2018b; Fianu et al. 2021, 2024; Su and Vos 2024). From such structures, it is clear that DSIF acts as “cement” for the ternary elongation complex by forming an extensive network of interactions with the surface of RNAPII, DNA, and the nascent RNA (Fig. 1B). DSIF quite literally “clamps” the emerging upstream DNA and the nascent RNA, indicating how RNAPII can remain stably attached to its template upon DSIF binding. Meanwhile, the main structured module of NELF binds to the front bottom surface of RNAPII with two long flexible “tentacles” that are dynamic and/or unstructured and hence escape resolution by cryo-EM. Intriguingly, small conserved domains lie at the distal end of each of the tentacles, including an RNA recognition motif (RRM) at the end of the NELF-E subunit that is required for the inhibitory effect of NELF on transcription (Yamaguchi et al. 1999). Importantly, the first structures of RNAPII–DSIF–NELF captured the complex in a “paused” conformation, where RNAPII is likely arrested due to the active site being in a configuration incapable of incorporating nucleotides. Here, NELF adopts a closed conformation, blocking access of TFIIS to the RNAPII funnel and hence inhibiting transcription reactivation in the event of backtracking (Vos et al. 2018b). Further studies revealed an additional “poised” conformation, where the RNAPII active site is pretranslocated and thus is in a transcriptionally active conformation. Here, NELF adopts an open structure that permits TFIIS binding (Su and Vos 2024). This suggests that a key function of NELF conformation switching is in determining TFIIS accessibility and thus the commitment of RNAPII to reactivation versus backtracking/arrest. This idea is strongly supported by recent structures of the pausing complex in the context of a nucleosome that capture NELF in its respective open and closed conformations but now indeed are associated with backtracked RNAPII (Naganuma et al. 2025).

## Events at the promoter-proximal checkpoint

**Figure 2.**
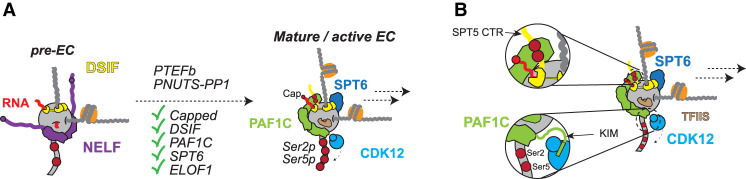
Maturation of the elongation complex and transcription activation. (*A*) The inactive, paused complex must be converted into a transcriptionally competent elongation complex through a series of steps. These include modification of the nascent pre-mRNA 5′ end by capping and loading of key elongation factors such as DSIF, PAF1C, SPT6, and ELOF1. Activation depends on the kinase and phosphatase activities of P-TEFb and PNUTS–PP1, respectively, though the specific targets of PNUTS–PP1 are unknown. One important function of the PAF1 complex is to activate CDK12/13 for phosphorylation of RNAPII Ser2 and Ser5 residues. (*B*) PAF1C is a multimodal stimulator of productive transcript elongation, first through the binding of PAF1C (subunit RTF1) to the phosphorylated C-terminal repeat domain of SPT5 (RTF1 is a strong allosteric stimulator of transcription), and second by the direct activation of CDK12 by the cyclin K-interacting motif (KIM) of the CDC73 subunit.

A complex series of events occurs between transcriptional initiation, the promoter-proximal pause, and pause release—the point at which a matured RNAPII complex begins productive elongation (Fig. 2A). Failure to execute any of these early steps may theoretically prevent passage into productive elongation and flag a failure of RNAPII to pass the PPC. Here we divide the types of reactions involved in elongation complex assembly into four categories but stress that although the chronology of some events is well defined, in the majority of cases it is not, and some processes could be occurring either stepwise or in parallel.

### Phosphorylation events

Progress through the transcription cycle is governed by phosphorylation of the C-terminal domain (CTD) of the largest RNAPII subunit, RPB1. In humans, the CTD contains 52 repeats of a heptapeptide comprising Tyr1–Ser2–Pro3–Thr4–Ser5–Pro6–Ser7 that is phosphorylated by protein complexes containing kinases such as TFIIH (CDK7), P-TEFb (CDK9), and CDK12 and CDK13 with their cognate cyclins (Pluta et al. 2023). RNAPII exists in two major forms in cells: a largely unphosphorylated form (RNAPIIA [IIA]) and a hyperphosphorylated form (IIO). The conversion from IIA to IIO happens through a series of well-studied CTD phosphorylation and dephosphorylation steps, the first of which is phosphorylation at serine 5 (Ser5) residues by the CDK7/cyclin H module of TFIIH during transcription initiation (Feaver et al. 1991, 1994; Lu et al. 1992; Roy et al. 1994). This results in dissociation of the Mediator complex and severs the connection to the general transcription factors of the preinitiation complex (Svejstrup et al. 1997; Søgaard and Svejstrup 2007; Wong et al. 2014; Velychko et al. 2024). Upon promoter escape, RNAPII then associates with DSIF and NELF at the promoter-proximal pause. This paused, serine 5 phosphorylated RNAPII (named IIPP) intermediate is extremely short-lived, as it cannot be detected in normal cells. However, recent analysis showed a striking stabilization of IIPP (as an intermediate between IIA and IIO) in cells deficient for the Integrator subunit INTS6 (Blears et al. 2024). At and beyond the PPC, the CTD undergoes further phosphorylation at both Ser5 and Ser2, eventually reaching its fully hyperphosphorylated (IIO) form as it transitions into productive elongation. Phosphorylation at the promoter-proximal pause is performed by P-TEFb (CDK9), with the CDK12/13–cyclin K complex playing a key role later in the full maturation and maintenance of the elongation complex (Fan et al. 2020; Lopez Martinez et al. 2025).

Interestingly, protein phosphatase 2A (PP2A) is recruited to promoter-proximal paused RNAPII complexes through an interaction with Integrator (Huang et al. 2020; Vervoort et al. 2021). Structural work indicates that PP2A is positioned in proximity to the C-terminal domain of RNAPII (Fianu et al. 2021). Mutation or depletion of PP2A or certain Integrator subunits results in a general increase in both SPT5 and RNAPII CTD phosphorylation (Behre et al. 1999; Huang et al. 2020; Hu et al. 2021; Vervoort et al. 2021). Specific immunoprecipitation of Integrator-associated, paused RNAPII complexes from the chromatin of INTS6-deficient cells indicates that PP2A rather specifically removes TFIIH-mediated CTD phosphorylation at serine 5 (Blears et al. 2024). Such dephosphorylation is likely critical for the recycling of RNAPII back into the pool of free RNAPIIA forms, and thus CTD dephosphorylation might be specific for RNAPII complexes destined for premature termination by Integrator. However, an alternative possibility is that Integrator-associated PP2A always dephosphorylates RNAPII, even those elongation complexes that subsequently enter the productive elongation phase of transcription. In this scenario, P-TEFb would then rephosphorylate RNAPII that passes the checkpoint, this time possibly often at both Ser5 and Ser2 (see Lopez Martinez et al. 2025), releasing it into productive elongation.

In addition to RNAPII, P-TEFb also phosphorylates the C-terminal repeat (CTR) region of the DSIF subunit SPT5 (Yamada et al. 2006; Sun and Fisher 2025). Importantly, although DSIF initially mediates RNAPII pausing, it is also required for efficient, processive elongation, a function that is conserved in yeast and prokaryotes (Winston et al. 1984; Swanson et al. 1991; Li et al. 1992; Burova et al. 1995; Decker 2021). Therefore, as SPT5 plays a key role in both pausing and elongation, it requires conversion from a negative to a positive elongation factor at the promoter-proximal pause, brought about via its phosphorylation by P-TEFb. Two distinct phosphorylation patterns have been observed for SPT5. First, hyperphosphorylation of its C-terminal repeats (CTRs) occurs in a manner somewhat akin to phosphorylation of the RPB1 CTD repeats (Ivanov et al. 2000; Kim and Sharp 2001) and is required for the transcription stimulatory effect of (or suppression of pausing by) SPT5 (Fig. 2B; Yamada et al. 2006; Fong et al. 2022; Sun and Fisher 2025). Second, unbiased phosphoproteomic screening for P-TEFb kinase substrate targets led to identification of an additional phosphorylation site in SPT5: a specific residue (S666) found in a short flexible loop linking the KOWx and KOW4/5 domains that form the SPT5 RNA clamp (Sansó et al. 2016; Parua et al. 2020). Genome-wide studies show that mutation of this residue suppresses transcription and enhances pausing (Hu et al. 2021; Fong et al. 2022). Furthermore, P-TEFb may also phosphorylate the CTD linker region of RPB1 (distinct from CTD repeat phosphorylation), which likely serves to stabilize the interaction of RNAPII with elongation factor SPT6 (Vos et al. 2018a). In yeast, phosphorylation of the CTD linker region by Bur1 (the CDK9 homolog) is required for association of Spt6 with RNAPII in vivo (Sdano et al. 2017; Chun et al. 2019).

So, what happens when P-TEFb-mediated phosphorylation cannot occur? Recent proteomics analysis of the RNAPII interactome in DRB-treated cells provides an informative overview of the paused transcription complexes stabilized by DRB and hence the relevant factors working upstream of, or as an alternative to, P-TEFb activity (Blears et al. 2024). This includes a striking stabilization of RNAPII's interaction with P-TEFb, DSIF, and NELF (as expected) but also of its interaction with mRNA-capping enzymes as well as with factors such as ELOF1, whose precise roles in pausing and pause release are unknown.

In other examples of RNAPII phosphorylation or dephosphorylation, it is clear that a complex series of protein–protein interactions serves to position and activate transcriptional kinases or phosphatases to achieve specific and timely activity toward their protein substrates. This is in contrast to kinase regulation in the cell cycle, where CDKs such as CDK1 act on a plethora of substrates and are regulated primarily through the timely production (and degradation) of the cognate, cell cycle stage-specific cyclins that determine their targets. With transcription CDKs, which are constitutively associated with their cyclin, specific events such as RNAPII or SPT5 phosphorylation only occur once the correct protein–protein interactions, conformations, and enzymatic activation have been achieved. Indeed, activation of the CTD kinase activity of TFIIH (CDK7–cyclin H) is brought about by association with Mediator during initiation (Kim et al. 1994; Robinson et al. 2016; Abdella et al. 2021; Chen et al. 2021; Schilbach et al. 2023). Likewise, recent data show that the CDK12/CDK13 kinases (with cyclin K) are specifically activated by interaction with a domain in the CDC73 subunit of the elongation-specific polymerase-associated factor 1 complex (PAF1C) (Fig. 2B; Lopez Martinez et al. 2025). Interestingly, although P-TEFb/CDK9 can be activated by the HIV Tat protein (Schulze-Gahmen et al. 2013), exactly how it is activated in normal cells to specifically target promoter-proximally paused RNAPII complexes remains somewhat uncertain.

Efficient termination of transcription at the 3′ ends of genes, which occurs concurrently with mRNA cleavage and polyadenylation, requires the phosphatase activity of the PNUTS–PP1 complex to dephosphorylate SPT5. The model here is that PNUTS–PP1 deactivates SPT5, thereby slowing down transcription and leaving RNAPII as a “sitting duck” for removal by the termination machinery (Cortazar et al. 2019). Surprisingly, however, two recent studies uncovered what appears to be a phenotypically separate function of PNUTS–PP1 in regulating pause release at the beginning of genes (Kelley et al. 2024; Wang et al. 2024). In these studies, depletion of PNUTS–PP1 clearly restricts RNAPII to the promoter-proximal regions and reduces transcriptional output, suggesting that a PNUTS–PP1-mediated dephosphorylation event is required for negotiation of the PPC. The protein targets of this phosphatase activity have not yet been determined.

### Loading and exchange of elongation factors

RNAPII is the core component of a much larger protein cohort that regulates transcript elongation. During elongation, RNAPII thus dynamically associates with a variety of elongation factors to enable efficient and sustainable progression through the exceptionally long mammalian protein-coding genes and allow cotranscriptional processing of the nascent pre-mRNA transcript.

In the transition from pausing to elongation, interactions of paused RNAPII are disrupted to allow association with PAF1C, the binding of which appears to be mutually exclusive with NELF (Fig. 2A; Vos et al. 2018a,b, 2020). PAF1C is a multimodal stimulator of RNAPII transcription and integral to the transcript elongation process (Yu et al. 2015; Francette et al. 2021; Zumer et al. 2021; Qiu et al. 2023). Besides its effect on epigenetic modification, PAF1C's effect is achieved through allosteric activation of the RNAPII enzyme activity (which greatly increases transcription rates in vitro) (Vos et al. 2020) but also via the recently discovered role of the PAF1C subunit CDC73 in the activation of the CDK12/13 kinase activity described above (Lopez Martinez et al. 2025). Interestingly, based on what we know from the structures of the RNAPII–DSIF–PAF1C–SPT6 elongation complex (Vos et al. 2018a, 2020), the predicted CDC73–CDK12/cyclin K interaction places the kinase in the immediate vicinity of the RPB1 CTD emerging from the elongation complex (Fig. 2B; Lopez Martinez et al. 2025). Importantly, CDK12/13 activity (Dubbury et al. 2018; Fan et al. 2020; Tellier et al. 2020) and its direct stimulation by PAF1C/CDC73 (Lopez Martinez et al. 2025) are essential for sustained elongation through long coding regions. Hence, correct PAF1C loading is critical for the transition from promoter-proximal pausing to elongation and may thus constitute an important checkpoint criterion.

In addition to DSIF and PAF1C, elongation factors such as SPT6 and ELOF1 are also loaded onto RNAPII in order to form an active elongation complex. SPT6 association results in allosteric activation of the elongation complex through structural rearrangement of the RNAPII stalk and the RNA/DNA clamps of DSIF, thereby permitting translocation of RNAPII (Vos et al. 2018a). SPT6 also serves as a central regulator of RNAPII passage through chromatin by chaperoning nucleosomes and mediating cotranscriptional deposition of histone H3K36 methylation (Nojima et al. 2018; Zumer et al. 2021; Markert et al. 2025). ELOF1 stabilizes the elongation complex and is important for normal rates of transcript elongation (Geijer et al. 2021; van der Weegen et al. 2021; Dai et al. 2025; Wu et al. 2025). Together, DSIF, PAF1C, SPT6, and ELOF1 thus appear to form the core elongation machinery that enables processive transcription through long genes.

Reconstitution studies in vitro provided evidence that P-TEFb is able to stimulate the exchange of NELF for PAF1C and SPT6, converting a paused, transcriptionally restricted elongation complex into an activated, transcriptionally competent form (Vos et al. 2018a). Recent experiments using a chemical genetic system for functional dissection of SPT5 nicely complement these data in vivo (Ramanathan et al. 2016; Sun and Fisher 2025). Importantly, the manner in which DSIF, PAF1C, and SPT6 interact with RNAPII suggests how phosphorylation events may lead to pause release. For example, besides RPB1 CTD phosphorylation, the RTF1 subunit of PAF1C interacts directly with the phosphorylated CTR repeats in DSIF (Vos et al. 2020), whereas SPT6 interacts with the phosphorylated CTD linker (Vos et al. 2018a), and phosphorylation of S666 in the KOWx–KOW4/5 linker of SPT5 might directly alter the interaction of the complex with the nascent RNA. These are all potential mechanisms through which P-TEFb or other kinases may bring about checkpoint licensing and transcription activation through factor exchange.

### Pre-mRNA capping and modification

All eukaryotic mRNAs are processed by the addition of a cap structure to the RNA 5′ end, which is formed in a stepwise fashion by capping factors RNGTT and CMTR1 through removal of the γ-phosphate of the terminal nucleotide and addition of 7-methylguanosine, followed by 2′-hydroxymethylation of the second nucleotide to form m^7^GpppX^m^-RNA. The cap is a fundamental feature of the mRNA, protecting it from exonucleases and acting as a unique recruitment point for proteins involved in pre-mRNA splicing, polyadenylation, nuclear export, and translation (Ramanathan et al. 2016). The cap interacts with the cap binding complex (CBC) to form a mature and translatable mRNA ribonucleoprotein (mRNP), and the CBC makes several connections with pausing factors to regulate early transcription events (Rambout and Maquat 2020).

Recruitment of the capping enzyme is dependent on, and stimulated by, CTD Ser5 phosphorylation (Cho et al. 1997; Ho and Shuman 1999; Schroeder et al. 2000; Schwer and Shuman 2011), and addition of the cap itself occurs early on in transcription at a distance downstream from the start site similar to that of promoter-proximal pausing and association with DSIF and NELF (Rasmussen and Lis 1993; Rambout and Maquat 2020; Garg et al. 2023). Importantly, numerous connections and dependencies between capping and pausing factors DSIF (Wen and Shatkin 1999; Pei and Shuman 2002; Mandal et al. 2004) and NELF (Schulze and Cusack 2017; Aoi et al. 2020) have been uncovered, again pointing to a close connection between promoter-proximal pausing and mRNA capping. Indeed, the stable population of short pre-mRNAs (i.e., those derived from promoter-proximally paused RNAPII) is generally capped (Salditt-Georgieff et al. 1980; Tamm et al. 1980; Nechaev et al. 2010).

Recent biochemical and structural studies of the mammalian capping process outline how the capping enzymes interact with the RNAPII surface and are positioned to enable processing of the nascent RNA as it emerges from the RNA exit channel (Noe Gonzalez et al. 2018; Garg et al. 2023; Li et al. 2024). The fact that these enzymes are directly tethered to RNAPII and the RNA exit channel provides another indication that capping in fact occurs immediately upstream of or at the promoter-proximal pause site. Intriguingly, experiments in yeast indicate that the human RNGTT homolog Ceg1 is released from RNAPII early in elongation in a manner dependent on the CTD phosphatase Fcp1 (Schroeder et al. 2000). Whether a similar release of human RNGTT is triggered by CTD dephosphorylation by either Integrator-associated PP2A or PP1–PNUTS at the PPC remains unclear.

Besides capping, pre-mRNA is cotranscriptionally modified in a number of other ways (Gilbert and Nachtergaele 2023). For example, N6-methyladenosine has emerged as a prevalent mRNA modification in eukaryotes. Intriguingly, depletion of the m^6^A methyltransferase Mettl3 in flies affects RNAPII pause release and nascent RNA transcription. Conversely, tethering Mettl3 to a gene promoter increases RNAPII pause release in a manner dependent on its m^6^A catalytic domain (Akhtar et al. 2021). Together, these examples indicate that not only assembly of the RNAPII elongation complex but also pre-mRNA modification occurs within the context of promoter-proximal pausing.

## The +1 nucleosome as a physical barrier at promoter-proximal pause sites

**Figure 3.**
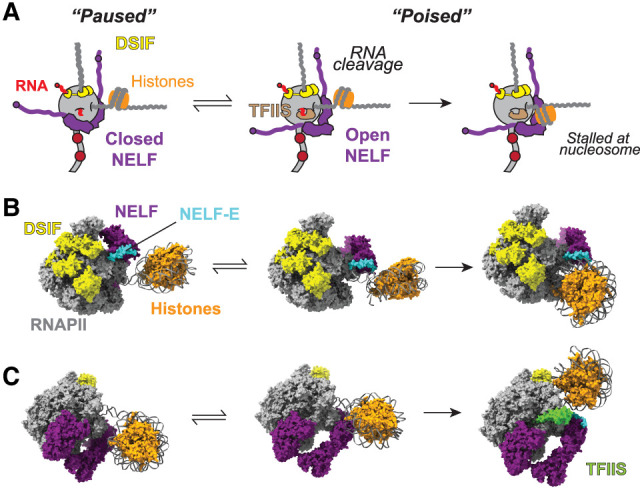
NELF, backtracking, and the +1 nucleosome cooperate in instigating promoter-proximal pausing. (*A*) Interactions of RNAPII–DSIF–NELF with the +1 nucleosome give rise to various stable arrangements. First, the +1 nucleosome can induce backtracking, and backtracked RNAPII is arrested with NELF in two distinct conformations, termed closed and open for simplicity. Closed NELF blocks recruitment of TFIIS, thereby inhibiting transcript cleavage and RNAPII reactivation. Open NELF is compatible with TFIIS binding and reactivation. In the presence of TFIIS, RNAPII can transcribe into the +1 nucleosome, partially unwrapping DNA from histones and eventually stalling at the SHL(−5) position (nucleosome entry site). (*B*) Corresponding cryo-EM structures (9J0O, 9J0P, and 9J0N). (*Left*) Backtracked, closed NELF. NELF-E is distal to the downstream DNA. (*Middle*) Backtracked, open NELF. In the open position, NELF-E is now proximal to and forms direct contacts with DNA. These contacts are required for optimal pausing in the presence of the nucleosome. (*Right*) Active (not backtracked) NELF transcribed to nucleosome entry site and bound by TFIIS. NELF-E binds directly to nucleosomal DNA. (*C*) The same structures as in *B* are shown in a different orientation, showing the binding of TFIIS. The closed NELF conformation blocks the binding of TFIIS, whereas the open conformation is permissive to TFIIS binding.

The first (+1) nucleosome downstream from the transcription start site represents a physical barrier that resists the forward motion of RNAPII (Kulaeva et al. 2013; Teves et al. 2014). In vitro, RNAPII is capable of transcribing through the nucleosome (Lorch et al. 1987), but nucleosomes are inhibitory (Izban and Luse 1992), and RNAPII undergoes pausing at distinct positions as DNA is unwrapped from the histones (Studitsky et al. 1997; Kireeva et al. 2005; Kujirai et al. 2018). Meanwhile, efficient passage through nucleosomes in vivo depends on elongation factors including histone chaperones such as SPT6 and FACT but also TFIIS, which enables RNAPII to overcome energetic barriers during transcript elongation through nucleosomes by stimulating transcriptional reactivation of the backtracked polymerase (Kulaeva et al. 2013; Teves et al. 2014). It is apparent that the +1 nucleosome represents a particularly challenging obstacle, which may help explain the importance of assembling a proficient elongation complex at the promoter-proximal pause prior to passing this nucleosome (Fig. 3). Interestingly, TFIIS is required for efficient pause release (Adelman et al. 2005; Sheridan et al. 2019), and NELF can inhibit transcript cleavage by TFIIS in vitro (Palangat et al. 2005) and greatly restricts the ability of TFIIS to allow transcription through a nucleosome (Naganuma et al. 2025). This suggests that NELF and the nucleosome cooperate to enhance the transcriptional barrier, which opens the possibility that one of the ways that NELF maintains the paused RNAPII complex is by somehow inhibiting or restricting reactivation of a backtracked RNAPII.

NELF-mediated RNAPII pausing occurs in exceptionally close proximity to the +1 nucleosome, and in the absence of NELF, RNAPII transcribes forward and becomes stuck in a partially unwrapped nucleosome (Aoi et al. 2020). Further insights into how RNAPII and NELF interact with the +1 nucleosome have come from recent biochemical structural studies (Fig. 3B,C; Naganuma et al. 2025). Remarkably, NELF blocks transcription of RNAPII into a +1 nucleosome at least partially through direct steric clashing but also through interactions between NELF-E and the nucleosomal DNA. RNAPII may then backtrack and become arrested. Importantly, TFIIS binds to this RNAPII–NELF complex and enables RNAPII progression to the nucleosome entry site, helping explain the role of TFIIS in pause release (Fig. 3B,C). Together with earlier work (Vos et al. 2018b), the recent study by Naganuma et al. (2025) suggests that there are two states of RNAPII–NELF in cells. One is paused and backtracked upstream of the +1 nucleosome with NELF in a closed conformation, whereas the other, with the cooperation of TFIIS through transcript cleavage, is poised at the nucleosome entry site with NELF in an open conformation. Together, these studies provide a structural and conceptual basis for how backtracking and conformational changes in NELF could instigate and determine pausing and release in relation to the +1 nucleosome (Fig. 3B,C).

One intriguing possibility is that NELF is not merely a pausing factor restricting transcription but that it also facilitates correct assembly of the elongation complex at the promoter-proximal pause site so that, without this function, RNAPII is released prematurely and is poorly equipped for nucleosome passage. Alternatively, NELF loss might affect +1 nucleosome stability through misregulation of nucleosome remodeling, an idea supported by the findings that first drew connections between RNAPII pausing, NELF, and nucleosome organization in promoter-proximal regions (Gilchrist et al. 2008, 2010). In this scenario, changes in ATP-dependent remodeler activity brought about by the pausing complex could influence the stability of the +1 nucleosome and hence the efficiency at which RNAPII can transcribe through it. ATP-dependent remodeling of nucleosomes to enable entry into gene bodies has typically been thought of in the context of gene regulation, as a means of controlling gene activity. However, it seems possible that specific control of +1 nucleosome stability is a key, constitutive determinant of pausing and release, which may be influenced by DSIF, NELF, Integrator, or other PPC regulators. Interestingly, a recent study indeed provided evidence that the BAF chromatin remodeler dynamically unwraps and evicts nucleosomes at accessible chromatin regions and that promoter-proximally paused RNAPII enhances the chromatin occupancy by BAF, specifically enhancing ATP-dependent +1 nucleosome eviction by this chromatin remodeler (Brahma and Henikoff 2024).

Histone modification is an integral feature of eukaryotic chromatin and transcription. Histone H3K4 methylation (H3K4me) is enriched in promoter regions, including at the first nucleosome, whereas H3K36 methylation is enriched in gene bodies (Hyun et al. 2017). Recent structural work showed how histone H3K36 trimethylation (H3K36me3) can be deposited by the RNAPII elongation complex and the SETD2 methyltransferase, providing a striking example of cotranscriptional histone modification (Markert et al. 2025). Whether H3K4 methylation is similarly deposited cotranscriptionally by the SET1/COMPASS complex at the +1 nucleosome remains to be determined, but recent data indicate that H3K4me3 modification constitutes a key step in the promoter-proximal pausing and release (Wang et al. 2023). Indeed, loss of H3K4me3 modification (but not H3K4me1 and H3K4me2) through rapid depletion of subunits of the SET1/COMPASS histone methyltransferase has significant consequences for transcription, specifically by stabilizing paused RNAPII and inhibiting its entry into elongation. These effects can be reversed by deletion of the relevant histone demethylases (KDM5A/B), showing that the effect is indeed through H3K4 methylation changes (Wang et al. 2023). Intriguingly, loss of H3K4me3 modification also resulted in a loss of the Integrator RNA cleavage module (INTS11 in particular) from paused RNAPII, and the polymerases that do escape the pause show decreased elongation rates (Wang et al. 2023), as observed in many other cases of Integrator loss (Aoi et al. 2020, 2021; Lykke-Andersen et al. 2021; Stein et al. 2022; Blears et al. 2024). Moreover, loss of H3K4me3 was also observed after NELF depletion (i.e., disruption of RNAPII pausing), supporting the link between pausing and methylation of the +1 nucleosome (Santana et al. 2024).

## Removal of immature transcription complexes—execution of the promoter-proximal checkpoint

**Figure 4.**
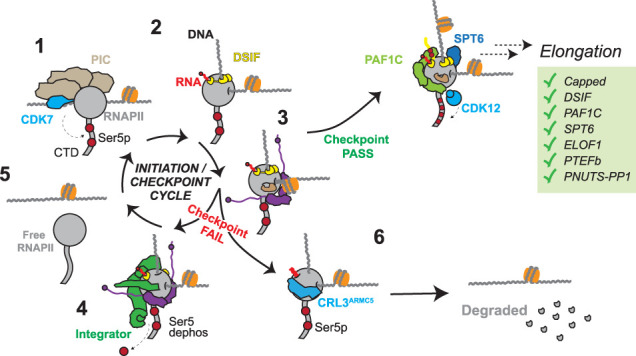
The promoter-proximal checkpoint. (1) RNAPII associates with the general transcription factors, forming the preinitiation complex. (2, 3) RNAPII initiates transcription and associates with DSIF and NELF. (*Top right*) At this point, a fully competent elongation complex may be formed, passing the checkpoint requirements and resulting in transcript elongation. Alternatively, if the checkpoint is failed, RNAPII either is dissociated by Integrator (4, 5), recycling RNAPII for new transcription, or is ubiquitylated and degraded in a CRL3^ARMC5^-dependent manner (6).

**Figure 5.**
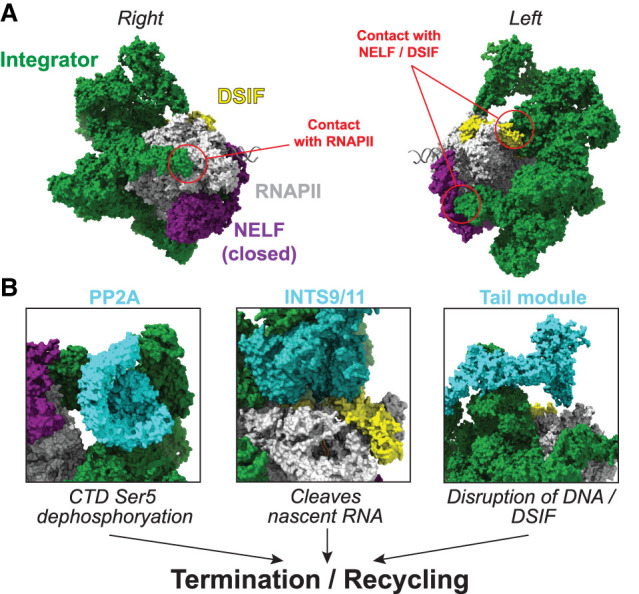
Integrator terminates paused RNAPII. (*A*) Cryo-EM structure of RNAPII–DSIF–NELF–Integrator (PDB: 8RBX). Integrator is a large (>1.5 MDa) complex that forms multiple contacts with RNAPII as well as with DSIF and NELF, specifically. The structure contains NELF in a closed conformation in which NELFB makes direct contact with Integrator. (*B*) Integrator executes several functions that together may enable transcription termination. (*Left*) Subunits INTS11/INTS9 bind at the RNA exit channel, positioned for cleavage of the emerging nascent RNA. (*Middle*) PP2A is positioned proximal to the CTD of RNAPII and dephosphorylates serine 5 residues. (*Right*) The tail module INTS10/INTS13/INTS14 is positioned so that it would clash with upstream DNA. (*C*) Schematic of termination and recycling of the paused elongation complex by Integrator to leave free, unphosphorylated RNAPII.

The PPC can be defined as a stage in the transcription cycle at which the cell examines biochemical and biophysical cues and decides whether or not to move forward with transcription (Fig. 4). So, what happens when the molecular processes fail to meet the requirements of the checkpoint? As should be evident from the descriptions above, Integrator constitutes one key mechanism of removing RNAPII that has failed to fulfill the various checkpoint criteria within a given time frame. Cryo-EM structures (Fig. 5A) show how Integrator can form a stable complex with RNAPII/DSIF/NELF, initially with 13 visible Integrator subunits (Fianu et al. 2021) but with the subsequent resolution of additional subunits (Fianu et al. 2024). Importantly, Integrator binds Pol II and the pausing factors DSIF and NELF in a manner that prohibits binding of SPT6 and PAF1C. It also binds the RPB1 CTD and positions PP2A to modulate RNAPII phosphorylation and elongation. Integrator then likely uses its RNA cleavage activity to dismantle the RNAPII elongation complex and recycle the polymerase for transcription (Fig. 5B). Integrator makes direct contacts with the surface of RNAPII, SPT5, and NELF-B, likely enabling it to specifically recognize promoter-proximally paused RNAPII (Fianu et al. 2021, 2024). The RNA cleavage module of Integrator (INTS4, INTS9, and INTS11), which is structurally similar to the RNA cleavage module of cleavage and polyadenylation specificity factor (CPSF) (Baillat et al. 2005; Pfleiderer and Galej 2021), is positioned to cleave RNA as it emerges from the RNAPII exit channel (Fianu et al. 2021, 2024). Interestingly, Integrator was found to primarily degrade RNA by an exonuclease-like activity in vitro rather than by producing distinct RNA cleavage products (Fianu et al. 2024). In contrast, catalytic inactivation of the cleavage subunit INTS11 inhibits the production of distinct, short RNA transcripts in vivo in *Drosophila melanogaster* (Tatomer et al. 2019).

The Integrator “tail” module (INTS10, INTS13, INTS14, and INTS15) is the latest addition to the Integrator structure, which is resolved in the RNAPII–Integrator structure after the introduction of the INTS15 subunit (Fianu et al. 2024). Intriguingly, the tail module contains a heterodimer of INTS13/INTS14 with structural similarity to the Ku proteins (Sabath et al. 2020) that bind double-stranded DNA ends (Mimori and Hardin 1986; Walker et al. 2001), and it has indeed been demonstrated that INTS13/INTS14 has nucleic acid binding capacity, which might contribute to the RNAPII termination process (Sabath et al. 2020). In the Integrator structure, these domains are positioned so that they practically clash with emerging upstream DNA, and it was proposed that this, together with destabilization of the upstream DNA clamp of DSIF, might facilitate RNAPII dissociation (Fig. 5B; Fianu et al. 2024). In fact, the NGN and KOW1/2 domains of SPT5 that form its DNA clamp are not resolved in the structure of RNAPII/DSIF/NELF/Integrator (Fianu et al. 2021, 2024) but are fully resolved in the structure without Integrator (Vos et al. 2018b). Hence, addition of Integrator appears to result in some destabilization of the SPT5 DNA clamp that might help facilitate termination.

However, Integrator is not the only mechanism by which RNAPII can be removed from DNA. Indeed, assessment of the chromatin-associated RNAPII interactome upon disruption of Integrator activity by loss of the INTS6 subunit shows increased association of paused RNAPII with a variety of other factors with the characteristics of transcription termination factors (Blears et al. 2024), suggesting that they may compensate for Integrator loss but possibly also contribute even in its presence. Among these were Senataxin, transcription termination factor 2 (TTF2), and PCF11. Another was ARMC5, a substrate recognition component of the Cullin 3–RING E3 ubiquitin ligase complex (CRL3^ARMC5^), which elicits the ubiquitin-directed degradation of the paused, serine 5 phosphorylated RPB1 (Fig. 4; Aoi et al. 2021, 2025; Lao et al. 2022; Blears et al. 2024; Cacioppo et al. 2024). Interestingly, not only does CRL3^ARMC5^ mediate increased ubiquitylation/degradation of RNAPII when Integrator is lost, but a substantial fraction of RNAPII is degraded in an ARMC5-dependent manner even in unperturbed, steady-state conditions, with *ARMC5* knockout cells containing twice as much RNAPII compared with wild type (Fig. 5; Lao et al. 2022; Blears et al. 2024). This sizeable increase in steady-state RNAPII amount strongly suggests that degradation of promoter-proximally paused RNAPII must occur surprisingly frequently also in cells with Integrator activity. Importantly, although loss of INTS6/8 or ARMC5 individually has little effect on HEK293 cell viability, loss of both is lethal, suggesting that the ability to remove faulty RNAPII complexes at the PPC is essential (Blears et al. 2024; Cacioppo et al. 2024).

In agreement with the idea that ARMC5 targets “faulty” RNAPII complexes at the promoter-proximal pause, CRL3^ARMC5^-dependent RNAPII polyubiquitylation and degradation are greatly increased upon loss of SPT5 (Aoi et al. 2021, 2025; Blears et al. 2024) or the capping enzyme RNGTT (Blears et al. 2024). Intriguingly, ARMC5 knockout also increases the amount of uncapped nascent transcripts (Blears et al. 2024). This result supports the idea that the PPC plays an important role in ensuring that only RNAPII elongation complexes with the potential to produce functional RNAs leave the pause and that unconstrained escape from the checkpoint can lead to futile transcription.

## Why terminate?

Already in the 1970s, a number of studies suggested that a vast number of RNAPII transcripts are terminated prematurely (Tamm 1977; Fraser et al. 1978, 1979; Evans et al. 1979; Tamm and Kikuchi 1979; Salditt-Georgieff et al. 1980; Tamm et al. 1980), a finding that has since been confirmed by modern approaches (Krebs et al. 2017; Shao and Zeitlinger 2017; Steurer et al. 2018). A recent estimate suggests that as much as 90% of initiated transcription reactions may normally be terminated in the early phases (Steurer et al. 2018). One possibility is that this apparent inefficiency evolved for the purpose of gene regulation to enable control of transcription output. However, it is clear that the mechanisms involving the promoter-proximal pause, NELF, and Integrator are constitutive and occur to some extent on every protein-coding gene in metazoans. An alternative, arguably more likely explanation is therefore that the assembly of a productive elongation complex is a complex, multistep process, with various nonproductive, dead-end transcription complexes constantly being formed. Reversal to functional intermediates might not be possible or straightforward, meaning that removing the dead-end complexes and starting anew may be biologically preferable or inevitable for the overall speed and accuracy of the elongation complex assembly reaction and for avoiding long residence times of immobile elongation complexes at the beginning of genes. Indeed, experiments with cells lacking Integrator support the idea that such stabilized complexes may interfere with genome stability; for example, through clashing with DNA replication, prolonged periods of single-stranded DNA exposure, or the creation of R-loops (Bhowmick et al. 2023; Xu et al. 2023). The fact that cells have even evolved the CRL3^ARMC5^-dependent pathway to degrade RNAPII at the promoter-proximal checkpoint underscores the intrinsic importance of enabling relatively fast turnover of the polymerase at the pause.

An important objective during elongation complex assembly is to ensure that only mature elongation complexes enter elongation. Indeed, loss of Integrator and ARMC5 not only prolongs the half-life of RNAPII at the promoter-proximal pause but also concomitantly results in the release into genes of RNAPII elongation complexes that are transcriptionally compromised, manifesting as elevated RNAPII pausing within genes (Blears et al. 2024). Loss of function of these factors thus results in the release of RNAPII with a decreased proficiency in transcript elongation and/or the production of nonfunctional mRNA (see, e.g., Lykke-Andersen et al. 2021; Stein et al. 2022; Blears et al. 2024).

Interestingly, recent data suggest that in the absence of Integrator, immature RNAPII elongation complexes released into genes not only are prone to slow elongation and premature termination as previously indicated (Lykke-Andersen et al. 2021; Stein et al. 2022; Blears et al. 2024) but also generate incomplete pre-mRNAs with retained introns (Baluapuri et al. 2025). This suggests that promoter-proximal pausing is also used to load and perhaps activate mRNA splicing factors. Retroelements within the retained introns were found to form double-stranded RNA (dsRNA) that triggers the integrated stress response (ISR) (Baluapuri et al. 2025). Intriguingly, mutations in INT subunits are associated with human diseases such as cancer, ciliopathies, and neurodevelopmental disorders (Wagner et al. 2023), and patient cells show dsRNA accumulation and ISR activation (Baluapuri et al. 2025). Similarly, ARMC5 is mutated in macronodular adrenal hyperplasia with Cushing's syndrome (Assié et al. 2013; Alencar et al. 2014; Faucz et al. 2014; Gagliardi et al. 2014; Elbelt et al. 2015). Together, these severe physiological consequences of defective PCC execution further emphasize the importance of ensuring correct elongation complex assembly at the beginning of protein-coding genes in metazoans.
